# The role of cannabidiol treatment in reducing HER2 expression and inducing ER stress-mediated apoptosis in HER2-positive breast cancer

**DOI:** 10.1186/s12906-026-05523-y

**Published:** 2026-08-13

**Authors:** Eser Çakmak, Bi̇rşen Bi̇lgi̇ci̇

**Affiliations:** Department of Medical Biochemistry, Faculty of Medicine, Ondokuz Mayıs University, Atakum/Samsun, 55139 Turkey

**Keywords:** Cannabidiol, Trastuzumab, HER2-positive breast cancer, ER stress, Apoptosis

## Abstract

**Background:**

Cannabidiol (CBD) has attracted attention as a potential anticancer agent due to its observed efficacy, particularly in breast cancer. However, the exact molecular pathway through which it exerts its anticancer effects remains unknown. The present study aimed to investigate the effects of CBD on the HER2 receptor, endoplasmic reticulum (ER) stress signaling pathway, and apoptosis in HER2-positive breast cancer cells, as well as to examine the effect of trastuzumab (TZB), used in treatment, on the ER stress signaling pathway.

**Methods:**

For this purpose, SKBR3 cells were treated with CBD at three different concentrations (9.38, 18.75, and 37.5 µM), TZB at 62.5 µg/mL, and two combinations of TZB and CBD (18.75 µM CBD + 62.5 µg/mL TZB and 37.5 µM CBD + 62.5 µg/mL TZB). Cell proliferation was assessed using real-time cell analysis, while expression levels of ER stress-associated genes were measured by qPCR. Apoptosis was analyzed by Annexin V/PI flow cytometry.

**Results:**

Findings revealed that CBD, TZB, or their combination were associated with reduced cell proliferation, significant activation of ER stress pathways, and increased apoptosis, while CBD was associated with marked downregulation of HER2 gene expression. Treatment with 9.38 µM CBD, 18.75 µM CBD, 37.5 µM CBD, 62.5 µg/mL TZB, 18.75 µM CBD + 62.5 µg/mL TZB, and 37.5 µM CBD + 62.5 µg/mL TZB significantly increased GRP78, PERK, IRE1, ATF6, eIF2A, ATF4, CHOP, and XBP1s gene expression levels, and significantly decreased HER2 gene expression at all doses except 62.5 µg/mL TZB. All doses significantly increased the total number of apoptotic cells and were associated with significantly reduced cell viability.

**Conclusions:**

The present findings demonstrated that both TZB and CBD are associated with activation of the ER stress response and induction of apoptosis in HER2-positive breast cancer cells, and that CBD is associated with downregulation of HER2 receptor gene expression. Collectively, our results indicate that CBD may have potential as an adjunct or alternative strategy in HER2-positive breast cancer, although further in vivo studies are required to evaluate the efficacy and safety of its combination with low-dose TZB.

## Background

Breast cancer (BC) constitutes a critical public health priority on a global scale. As of 2022, it was the most frequently diagnosed malignancy in women, accounting for approximately 2.3 million new cases [1]. Furthermore, BC surpassed all other cancers as the principal cause of female cancer mortality worldwide that year, exhibiting the highest incidence rates in 157 nations and the highest mortality rates in 112 countries [2]. Approximately 15% of breast cancers overexpress human epidermal growth factor receptor 2 (HER2), localized at 17q12 [3]. HER2, a member of the epidermal growth factor receptor (EGFR) family, plays a central role in signaling processes that control cell proliferation, differentiation, and survival [4]. HER2 overexpression is associated with metastasis, invasion, and a poor prognosis, and is also a predictor of response to anti-HER2-targeted therapies [5]. Recent studies have focused on the regulation of cell growth, endoplasmic reticulum (ER) stress, the unfolded protein response (UPR) signaling pathway, and apoptotic cell death associated with this pathway in the treatment of HER2-positive BC [6, 7].

In cancer pathogenesis, the dynamic interaction between ER stress and apoptosis is critical. Various genetic and metabolic abnormalities occurring in tumors create adverse microenvironments that lead to persistent ER stress in tumor cells, ultimately affecting their function, survival, and fate [8]. Meanwhile, the UPR, initiated by the binding of Glucose-Regulated Protein 78 kDa (GRP78) to unfolded proteins, attempts to restore homeostasis through major sensors such as PRKR-like ER Kinase (PERK), Inositol-Requiring Enzyme 1 (IRE1), and Activating Transcription Factor 6 (ATF6) [9]. Under prolonged or severe ER stress, the adaptive capacity of the UPR is exceeded, and signaling is initiated to promote apoptosis [10]. Specifically, the increase in C/EBP Homologous Protein (CHOP) expression via the PERK-Eukaryotic Translation Initiation Factor 2α (eIF2A)-Activating Transcription Factor 4 (ATF4) axis triggers cell death by activating mitochondrial apoptotic pathways [11].

The mechanisms of antitumor activity of trastuzumab (TZB), a monoclonal antibody, primarily involve preventing HER2 receptor dimerization, increasing endocytic degradation of the receptor, and activating the immune response through antibody- and/or complement-dependent cellular cytotoxicity [12, 13]. There is limited literature available on the relationship between TZB and ER stress in HER2-positive BC cells [6]. Although TZB is a relatively safe drug in HER2-positive BC [14], it has many side effects, including cardiotoxicity [15, 16] and pulmonary toxicity [17] as the most important effects, as well as gastrointestinal effects [18] and hepatotoxicity [19, 20]. Furthermore, resistance to TZB therapy poses a significant challenge in the management of these patients [21–23]. In HER2-positive BC, is there potential for combining low-dose TZB with Cannabidiol (CBD), an anticancer agent that has gained attention in recent years, as a therapeutic strategy to reduce TZB’s side effects and increase its efficacy? Or could CBD be an alternative monotherapy option to TZB?

Cannabidiol, a cannabinoid derivative derived from *Cannabis sativa* that does not exhibit psychotomimetic effects, is considered a potential therapeutic agent in inflammatory diseases, chronic neurodegenerative diseases, as well as various types of cancer [24–27]. Although the mechanisms underlying CBD’s antitumor effects are not yet fully elucidated, one of the leading hypotheses is that these effects occur through the activation of CB1 and CB2 receptors and interaction with the endocannabinoid system [28, 29]. However, studies over the past five years have shown that ER stress pathways play a significant role in the antitumor effects of CBD [30–35].

 In vitro studies in BC provide strong evidence that CBD reduces proliferation [36, 37], suppresses invasion [38], and induces apoptosis [27, 37, 39]. In vivo studies have also yielded results supporting these findings; in tumor-generated mouse models, CBD administration was reported to reduce tumor growth [40], invasion, and metastasis [39] significantly. Studies investigating the molecular basis of these anticancer effects of CBD have shown that CBD treatment significantly reduces the viability of BC cell lines and triggers ER stress, UPR activation [35]. In another study it was found that CBD increased apoptosis in a dose-dependent manner and induced ER stress via phospho-eIF2A, particularly in BC cells [41]. A recent study conducted with HER2-positive BC cells also reported that CBD increased the levels of ATF4 and CHOP proteins, which are important effectors of ER stress pathways [42]. Although these findings indicate that CBD exerts antitumor effects by modulating ER stress mechanisms across different BC subtypes, no comprehensive study in the literature has specifically investigated the anticancer effects of CBD via the ER stress signaling pathway in HER2-positive BC, which is known for its aggressive course and treatment resistance. Accordingly, this study aims to fill this important gap in the literature by investigating the potential anticancer effects of CBD in HER2-positive BC cells, its effects on the HER2 receptor, and the role of ER stress pathways in these effects. Another objective of the study is to evaluate the anticancer effect of TZB not only through HER2 receptor inhibition but also through its potential to induce ER stress. In addition, it is planned to determine the effects of CBD and TZB in combination on HER2 receptor and ER stress signaling pathways.

## Methods

### Cell culture

HER2-positive BC cell line SKBR3 was obtained from the American Type Culture Collection, and the cells were cultured in the 25 cm^2^-flasks of McCoy’s 5A modified medium (Gibco, cat. no. 16600-082) containing 10% fetal bovine serum (Gibco, cat. no. 10500-064), 1% L-Glutamine (Gibco, cat. no. 25030-081), 1% 100U penicillin/0.1 mg streptomycin (Gibco, cat. no. 15140-122) with CO_2_ 5% at 37^◦^C. The complete medium was removed when the cells reached 70-80% confluence, and they were irrigated with phosphate-buffered saline (PBS). After removal of PBS, the cells were removed by trypsin-ethylene diamine tetraacetic acid (EDTA;0.25% trypsin, 0.02% EDTA, Gibco, cat no. 25200-056). Then the cells were counted and 5 × 10^3^ cells/per well transferred into the E-plate for the proliferation-cytotoxicity assay. For the determination of gene expression and apoptosis by flow cytometry, cells were seeded in 25 cm^2^ flasks.

### Study groups

For RTCA analysis, two separate experimental sets were designed. The first set consisted of: (1) control, (2) 9.38 µM CBD, (3) 18.75 µM CBD, and (4) 37.5 µM CBD. The second set comprised: (1) control, (2) 18.75 µM CBD, (3) 62.5 µg/mL TZB, (4) 18.75 µM CBD + 62.5 µg/mL TZB, (5) 37.5 µM CBD, and (6) 37.5 µM CBD + 62.5 µg/mL TZB.

For gene expression and Annexin V/PI analyses, the following experimental groups were formed: (1) control, (2) 9.38 µM CBD, (3) 18.75 µM CBD, (4) 37.5 µM CBD, (5) 62.5 µg/mL TZB, (6) 18.75 µM CBD + 62.5 µg/mL TZB, and (7) 37.5 µM CBD + 62.5 µg/mL TZB. Only complete medium was added for the control group.

The CBD concentrations (9.38, 18.75, and 37.5 µM) were selected based on the effective dose range (5-30 µM) previously reported in SKBR3 cells [35, 42]. To confirm the biological relevance of these doses, a preliminary RTCA-based proliferation assay was performed with eight concentrations ranging from 75 µM to 0.29 µM, which yielded an IC50 value of approximately 27.9 µM, consistent with the cytotoxicity profile reported by Kim et al. [42]. The selected dose range thus encompassed the IC50 value and provided a meaningful window for combination studies. The TZB concentration (62.5 µg/mL) was chosen based on a previous study reporting an IC50 value of 63.25 µg/mL for TZB in SKBR3 cells [43].

### Real-time cell analyzer (RTCA)

SKBR3 cells were seeded at a density of 5 × 10³ cells/well into E-plates specifically designed for the xCELLigence RTCA system (Agilent Technologies, Inc.) and incubated at 37 °C with 5% CO₂ for 24 h. Subsequently, media containing the treatments were added to the respective wells. These treatments included CBD (Tocris Bioscience, Catalog #1570; purity ≥ 98% by HPLC, CAS Number 13956-29-1, PubChem ID 12302390) at three different concentrations (9.38, 18.75, and 37.5 µM), TZB (Roche) at 62.5 µg/mL, and two combinations of TZB and CBD (18.75 µM CBD + 62.5 µg/mL TZB and 37.5 µM CBD + 62.5 µg/mL TZB). 37.5 mM stock solution of CBD was prepared by dissolving 10 mg of CBD in 0.85 mL of DMSO. Further dilutions to the working concentrations were performed using cell culture medium. Control wells received complete medium without drugs. The E-plate was then monitored continuously for 72 h.

### Quantitative real-time PCR (RT-PCR)

After the 24th hour of drug administration, the medium was discharged from the flasks. Using the One Step-RNA Reagent (cat. no. BS410A Bio Basic Inc.) RNA isolation was performed. RNAs’ quality and quantity were evaluated by the Nanodrop 2000 spectrophotometer (Thermo Fisher Scientific, Inc.). Using the iScript™ cDNA Synthesis Kit (cat. no. 1708891; Bio-Rad Laboratories, Inc.), mRNAs were converted to cDNA according to the manufacturer’s protocol. Using the SsoAdvanced™ Universal SYBR Green Supermix (cat. no. 1725271; Bio-Rad Laboratories, Inc.) GAPDH, GRP78, PERK, IRE1, ATF6, eIF2A, ATF4, CHOP, XBP1s and HER2 genes expression were measured through qPCR, using the Biorad CFX 96 system (Bio-Rad Laboratories, Inc.) with the following cycling protocol: initial denaturation: 98˚C for 30 s; 40 cycles of [denaturation: 98˚C for 15 s, annealing/extension: 60˚C for 30 s]. The primer sequences of the genes are detailed in Table 1.

**Table 1:** Sequences of polymerase chain reaction primers

Gene	Forward primer, 5’→3’	Reverse primer, 3’→5’
GAPDH	GGAGCGAGATCCCTCCAAAAT	GGCTGTTGTCATACTTCTCATGG
GRP78	ACAATCAAGGTCTATGAAGGTGAAAGAC	CTCGAAGAATACCATTCACATCTATCTC
PERK	AATCATAGCTCCTTCACCACAAA	CATCTTCCACATCACAGTCTGTA
IRE1	CACAGTGACGCTTCCTGAAAC	GCCATCATTAGGATCTGGGAGA
ATF6	AGCATGTTCCTGAGGAGTTGG	AGGCTTATCTTCCTTCAGTGGC
eIF2A	TGGTGAATGTCAGATCCATTGC	TAGAACGGATACGCCTTCTGG
ATF4	ACGTTGGATGACACTTGTGATCTCTT	TGTAGTCTGGCTTCCTATCTCCTTCA
CHOP	GGAAACAGAGTGGTCATTCCC	CTGCTTGAGCCGTTCATTCTC
XBP1s	GCTGAGTCCGCAGCAGGT	CTGGGTCCAAGTTGTCCAGAAT
HER2	TGACACCTAGCGGAGCGAT	GGGGGATGTGTTTTCCCTCAA

### Annexin V/PI analysis

Detection of apoptosis was conducted using the Annexin V/PI apoptosis detection kit (Elabscience, ref E-CK-A211), according to the manufacturer’s protocol. After the 24th hour of drug administration, all 7 groups of cells were harvested by trypsinization, washed in PBS, and stained with Annexin V-FITC conjugate and propidium iodide. Cells were then analyzed by flow cytometry (BD FACSCalibur™; BD Biosciences) using BD CellQuest™ acquisition and analysis software (version 5.2.1; BD Biosciences).

### Statistical analysis

All experiments were performed in triplicate (*n* = 3). For gene expression analysis, the QIAGEN Relative Expression Software Tool (REST, 2009) was utilized. GAPDH was used as the housekeeping reference gene, and the control group was accepted as the calibrator. Flow cytometric analysis of Annexin V/PI staining was performed to assess cell death. Statistical analyses were carried out using SPSS software (version 25, IBM Corp., Armonk, NY, USA). The normality of the data distribution was tested with the Shapiro-Wilk test. As the data were normally distributed, comparisons among multiple groups were conducted using one-way analysis of variance (ANOVA), followed by Dunnett’s post hoc test to compare each treatment group with the control. Similarly, RTCA cell index values were also evaluated using one-way ANOVA, with post hoc comparisons performed using Tukey’s test. Values of *p* < 0.05 were considered statistically significant.

## Results

### CBD shows dose-dependent cytotoxic effects in SKBR3 cells and exhibits enhanced antiproliferative activity in combination with TZB

**Fig. 1:**
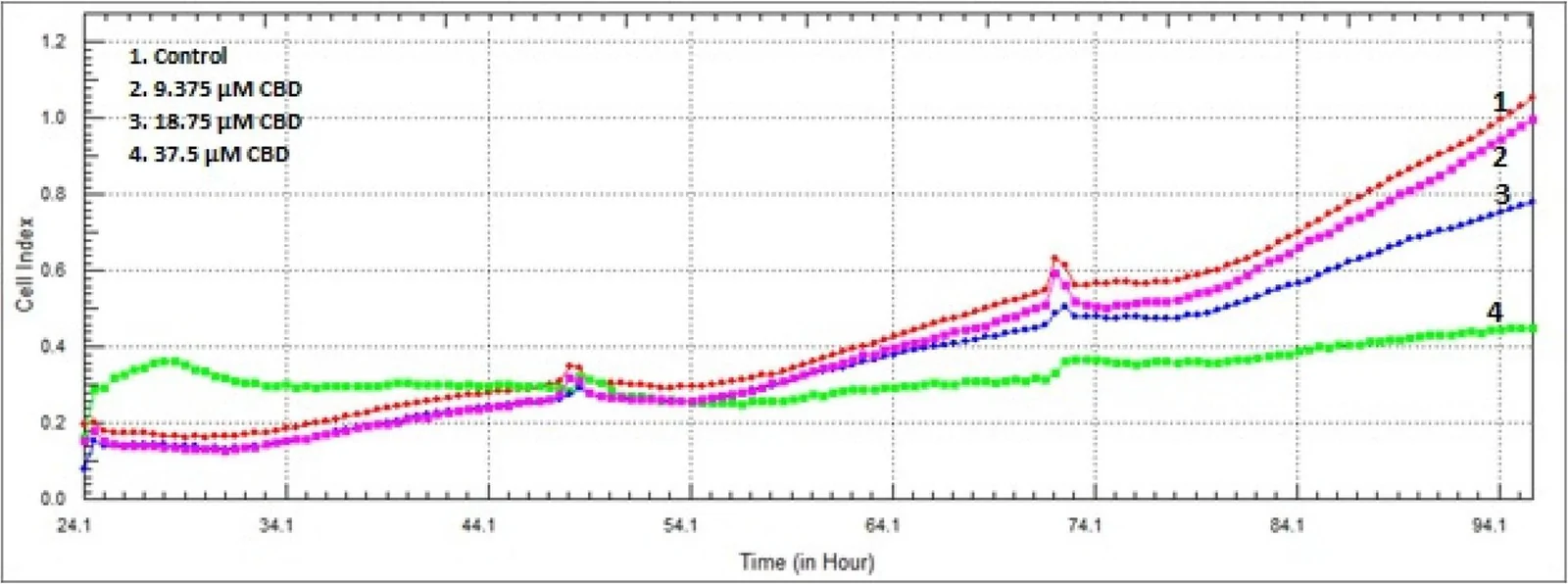
Real-time proliferation analysis of SKBR3 cells treated with increasing concentrations of CBD. SKBR3 cells were seeded in E-plates and allowed to adhere for 24 h, then treated with CBD at concentrations of 9.38 µM, 18.75 µM, and 37.5 µM for 72 h. Cell proliferation was monitored in RT using the xCELLigence RTCA system, and CI values were recorded at 15-minute intervals throughout the experiment. Data are presented as mean ± SD (*n* = 3). A dose-dependent decrease in CI was observed at 72 h, with significant reductions at 18.75 µM and 37.5 µM compared to control (*p* < 0.001), while the 9.38 µM dose showed no significant change (*p* > 0.05)

Findings from RTCA analysis, which allows real-time monitoring of cell proliferation and cytotoxicity, clearly demonstrated the dose-dependent cytotoxic effect of CBD in SKBR3 cells. At 72 h, low-dose CBD (9.38 µM) did not cause a significant change in cell index (CI) values compared to the control group, while a dose-dependent decrease in CI values was observed as the concentration increased (18.75 µM and 37.5 µM) (Fig. 1; Table 2).

**Table 2:** Cell Index values of SKBR3 cells treated with CBD doses for 72 hours. Data are presented as mean ± SD (*n*=3). Different superscript letters indicate significant differences between groups (*p* < 0.05, one-way ANOVA followed by Tukey's post-hoc test)

Groups (*n* = 3)	Control	9.38 µM CBD	18.75 µM CBD	37.5 µM CBD	*p*-value
Cell index mean ± SD	3.77 ± 0.17^a^	3.84 ± 0.13^b^	2.70 ± 0.11^c^	1.02 ± 0.1	0.000

^a^post-hoc Tukey control versus 18.75 µM CBD *p* = 0.000, control versus 37.5 µM CBD *p* = 0.000

^b^post-hoc Tukey 9.38 µM CBD versus 18.75 µM CBD *p* = 0.000, 9.38 µM CBD versus 37.5 µM CBD *p* = 0.000

^c^post-hoc Tukey 18.75 µM CBD versus 37.5 µM CBD *p* = 0.000

control versus 9.38 µM CBD *p* > 0.05

**Fig. 2:**
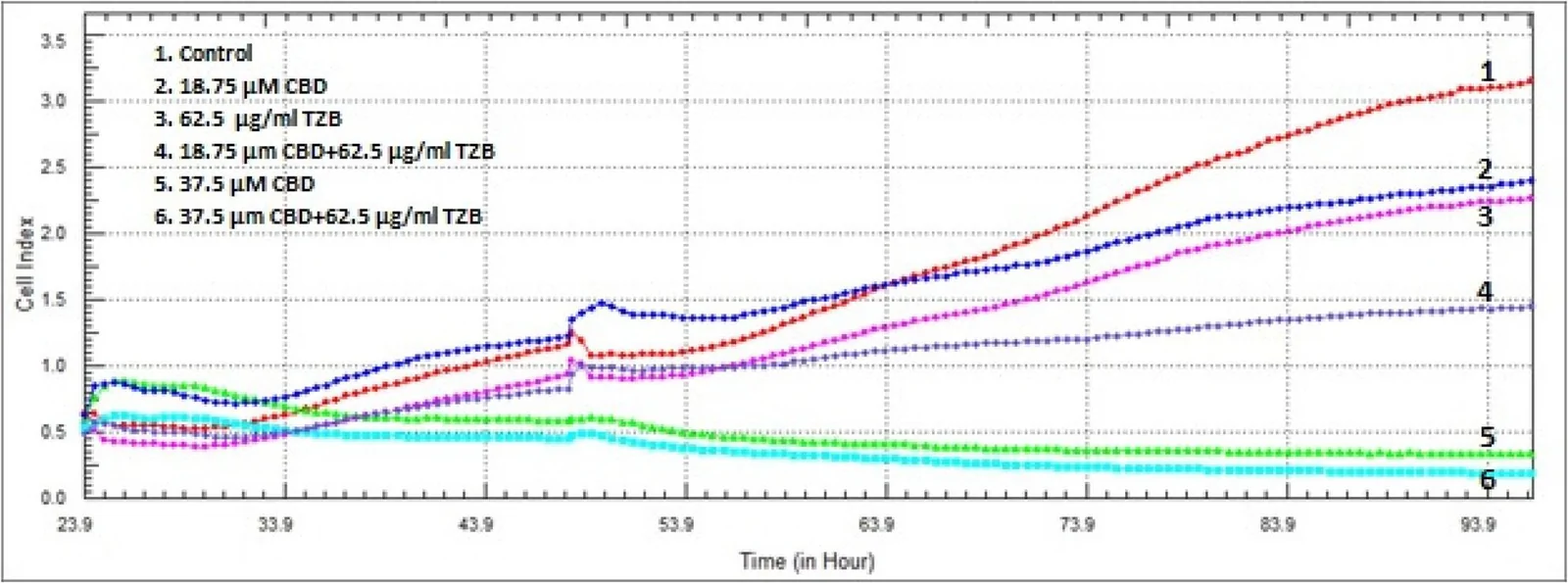
Real-time proliferation analysis of SKBR3 cells treated with CBD, TZB, and their combination. SKBR3 cells were seeded in E-plates and allowed to adhere for 24 h, then treated with CBD (18.75 µM and 37.5 µM), TZB (62.5 µg/mL), and their combinations for 72 h. Cell proliferation was monitored in real time using the xCELLigence RTCA system, and CI values were recorded at 15-minute intervals throughout the experiment. Data are presented as mean ± SD (*n* = 3). At 72 h, all treatment groups showed significant reductions in CI compared to control (*p* < 0.05), with the most pronounced decreases observed in the combination groups

While the application of 62.5 µg/mL TZB and 18.75 µM CBD individually showed similar cytotoxic effects, their combination exhibited markedly enhanced cytotoxicity. However, the combination of 37.5 µM CBD with 62.5 µg/mL TZB produced a cytotoxic effect comparable to that of 37.5 µM CBD alone, which already demonstrated high cytotoxicity (Fig. 2; Table 3).

**Table 3:** Cell Index values of SKBR3 cells treated with TZB, CBD, and their combinations for 72 hours. Data are presented as mean ± SD (*n*=3). Different superscript letters indicate significant differences between groups (*p* < 0.05, one-way ANOVA followed by Tukey's post-hoc test)

Groups (*n* = 3)	Control	18.75 µM CBD	62.5 µg/mL TZB	18.75 µM CBD + 62.5 µg/mL TZB	37.5 µM CBD	37.5 µM CBD + 62.5 µg/mL TZB	*p*-value
Cell index mean ± SD	3.14 ± 0.35^a^	2.39 ± 0.24^b^	2.26 ± 0.34^c^	1.45 ± 0.06^d^	0.34 ± 0.09	0.19 ± 0.03	0.000

^a^post-hoc Tukey control versus 18.75 µM CBD *p* = 0.016, control versus 62.5 µg/mL TZB *p* = 0.005, control versus 18.75 µM CBD + 62.5 µg/mL TZB *p* = 0,000, control versus 37.5 µM CBD *p* = 0.000, control versus 37.5 µM CBD + 62.5 µg/mL TZB *p* = 0.000

^b^post-hoc Tukey 18.75 µM CBD versus 18.75 µM CBD + 62.5 µg/mL TZB *p* = 0.004, 18.75 µM CBD versus 37.5 µM CBD *p* = 0.000, 18.75 µM CBD versus 37.5 µM CBD + 62.5 µg/mL TZB *p* = 0.000

^c^post-hoc Tukey 62.5 µg/mL TZB versus 18.75 µM CBD + 62.5 µg/mL TZB *p* = 0.013, 62.5 µg/mL TZB versus 37.5 µM CBD *p* = 0.000, 62.5 µg/mL TZB versus 37.5 µM CBD + 62.5 µg/ml TZB *p* = 0.000

^d^post-hoc Tukey 18.75 µM CBD + 62.5 µg/mL TZB versus 37.5 µM CBD *p* = 0.001, 18.75 µM CBD + 62.5 µg/mL TZB versus 37.5 µM CBD + 62.5 µg/mL TZB *p* = 0.000

62.5 µg/mL TZB versus 18.75 µM CBD and 37.5 µM CBD versus 37.5 µM CBD + 62.5 µg/mL TZB *p* > 0.05

### CBD and TZB induce upregulation of ER stress markers and their downstream targets in SKBR3 cells

**Fig. 3:**
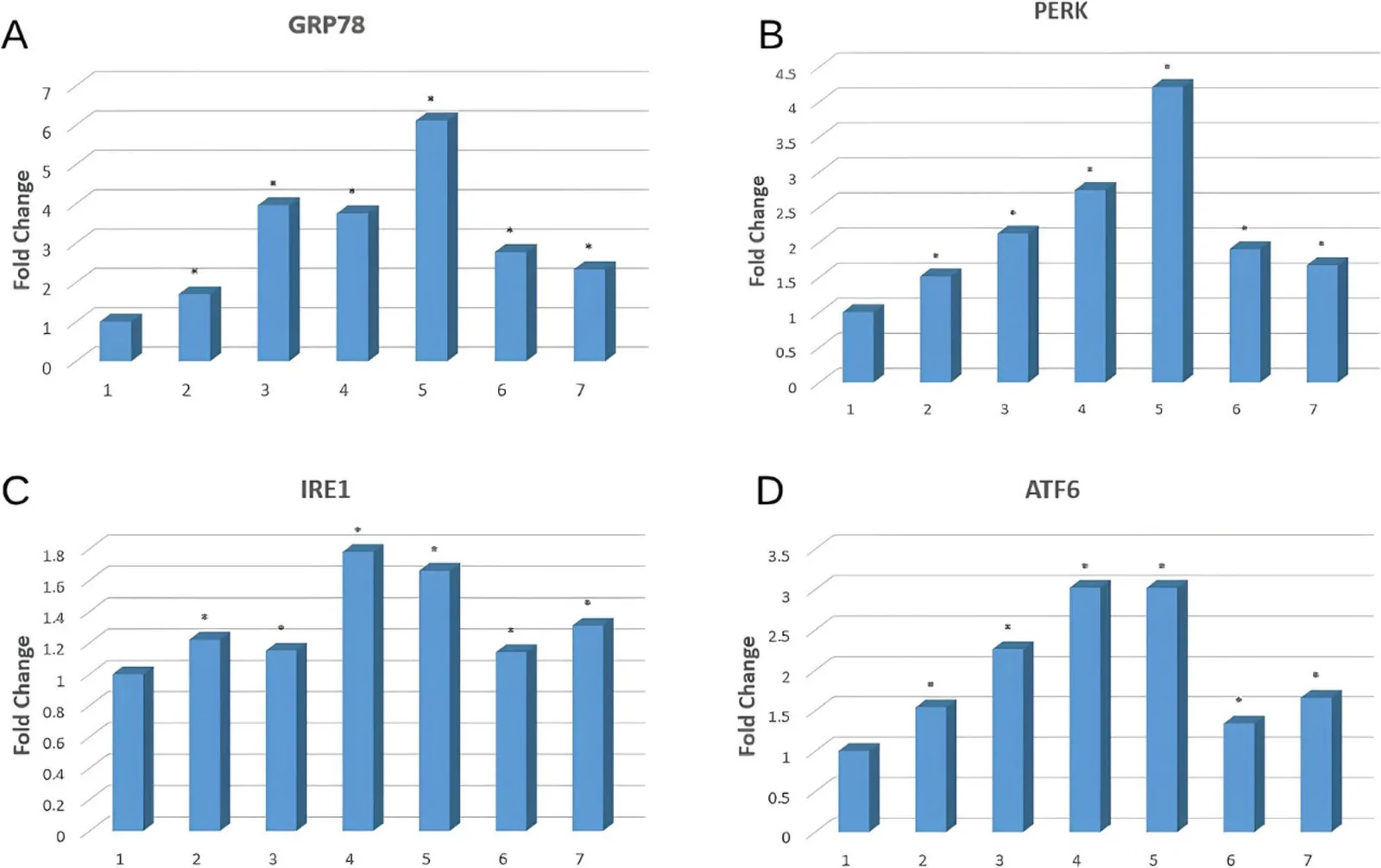
Effects of CBD and/or TZB on the relative mRNA expression of key ER stress markers in SKBR3 cells. SKBR3 cells were treated with CBD (9.38, 18.75, and 37.5 µM), TZB (62.5 µg/mL), and their combinations for 24 h. Relative mRNA expression levels of (A) GRP78, (B) PERK, (C) IRE1, and (D) ATF6 were determined by qPCR using GAPDH as the internal reference gene. Statistical analysis was performed using REST 2009 software with Pair-Wise Fixed Reallocation Randomization Test (2000 randomizations). *p < 0.05 indicates statistically significant differences compared to the control group. Detailed fold-change values, 95% confidence intervals, and exact p-values for all groups are provided in Table 4. (1) Control; (2) 9.38 µM CBD; (3) 18.75 µM CBD; (4) 37.5 µM CBD; (5) 62.5 µg/mL TZB; (6) 18.75 µM CBD + 62.5 µg/mL TZB; (7) 37.5 µM CBD + 62.5 µg/mL TZB

**Fig. 4:**
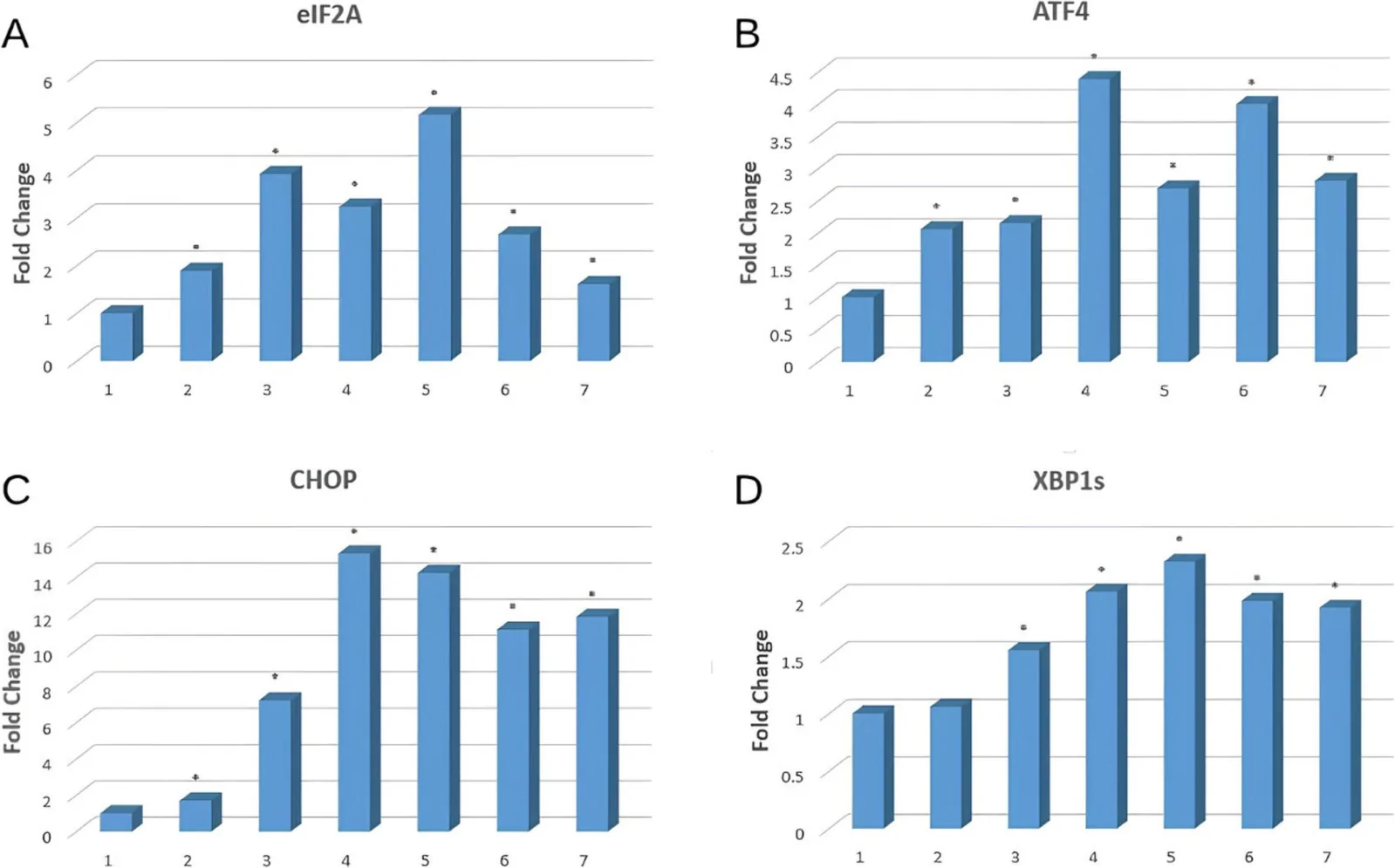
Effects of CBD and/or TZB on the relative mRNA expression of ER stress downstream target genes in SKBR3 cells. SKBR3 cells were treated with CBD (9.38, 18.75, and 37.5 µM), TZB (62.5 µg/mL), and their combinations (18.75 µM CBD + 62.5 µg/mL TZB and 37.5 µM CBD + 62.5 µg/mL TZB) for 24 h. Relative mRNA expression levels of (**A**) eIF2A, **B** ATF4, **C** CHOP, and **D** XBP1s were determined by qPCR using GAPDH as the internal reference gene. Statistical analysis was performed using REST 2009 software with the Pairwise Fixed Reallocation Randomization Test (2000 randomizations). *p < 0.05 was considered statistically significant compared to the control group. All fold-change values, 95% confidence intervals, and exact *p*-values for each gene and treatment group are provided in detail in Table 4. (1) Control; (2) 9.38 µM CBD; (3) 18.75 µM CBD; (4) 37.5 µM CBD; (5) 62.5 µg/mL TZB; (6) 18.75 µM CBD + 62.5 µg/mL TZB; (7) 37.5 µM CBD + 62.5 µg/mL TZB

In SKBR3 cells, consistent increases in protein/mRNA levels of major ER stress markers (GRP78, PERK, IRE1, ATF6) and their downstream targets (eIF2A, ATF4, CHOP, XBP1s) were observed across all experimental groups compared to the control group. These results demonstrate that CBD and TZB, both alone and in combination, activate ER stress pathways (Figs. 3 and 4; Table 4).

### CBD downregulates HER2 gene expression in a dose-dependent manner

**Fig. 5:**
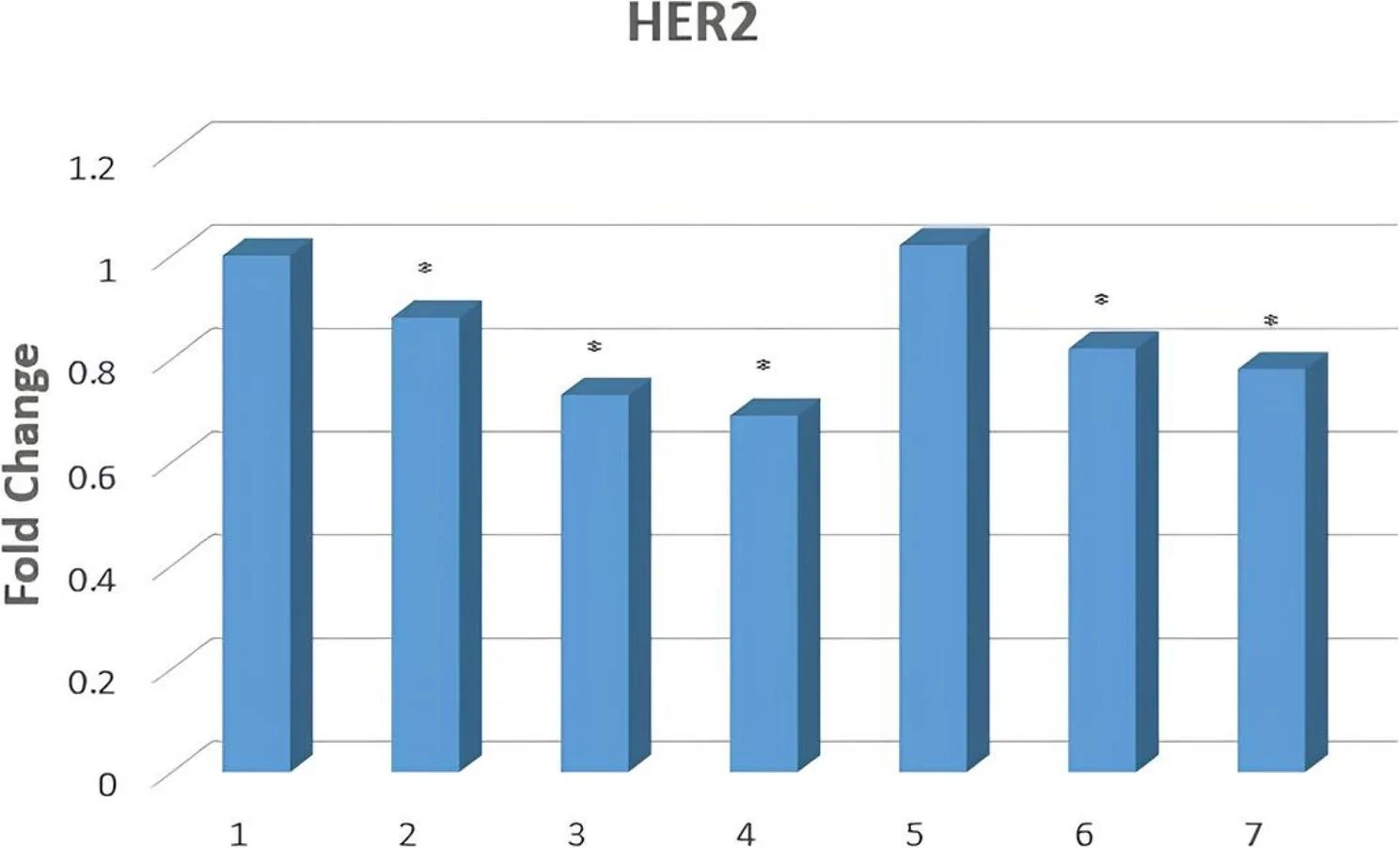
Effects of CBD and/or TZB on HER2 mRNA expression in SKBR3 cells. SKBR3 cells were treated with CBD (9.38, 18.75, and 37.5 µM), TZB (62.5 µg/mL), and their combinations (18.75 µM CBD + 62.5 µg/mL TZB and 37.5 µM CBD + 62.5 µg/mL TZB) for 24 h. Relative mRNA expression levels of HER2 were determined by qPCR using GAPDH as the internal reference gene. Statistical analysis was performed using REST 2009 software with the Pairwise Fixed Reallocation Randomization Test (2000 randomizations). *p < 0.05 was considered statistically significant compared to the control group. All fold-change values, 95% confidence intervals, and exact p-values for each gene and treatment group are provided in detail in Table 4. (1) Control; (2) 9.38 µM CBD; (3) 18.75 µM CBD; (4) 37.5 µM CBD; (5) 62.5 µg/mL TZB; (6) 18.75 µM CBD + 62.5 µg/mL TZB; (7) 37.5 µM CBD + 62.5 µg/mL TZB

Treatment with 9.38 µM CBD, 18.75 µM CBD, 37.5 µM CBD, 18.75 µM CBD + 62.5 µg/mL TZB, and 37.5 µM CBD + 62.5 µg/mL TZB significantly decreased HER2 gene expression levels by 0.88-fold (*p* = 0.018), 0.73-fold (*p* = 0.012), 0.69-fold (*p* = 0.000), 0.82-fold (*p* = 0.016), and 0.78-fold (*p* = 0.023), respectively. In contrast, 62.5 µg/mL TZB alone did not significantly alter HER2 expression (Fig. 5; Table 4).

**Table 4:** Effects of CBD and/or TZB on ER Stress Markers and HER2 Gene Expression in SKBR3 Cells Analyzed by REST

Group (*n* = 3)		GRP78	PERK	IRE1	ATF6	eIF2A	ATF4	CHOP	XBP1s	HER2
9.38 µM CBD	Fold-change	1.70	1.51	1.22	1.54	1.89	2.06	1.69	1.06	0.88
	95% Confidence Interval	1.420–2.032	1.299–1.746	1.180–1.264	1.391–1.699	1.820–1.976	1.699–2.497	1.664–1.722	0.993–1.124	0.854–0.902
	*p*	0.012	0.015	0.018	0.013	0.011	0.024	0.006	0.432	0.018
18.75 µM CBD	Fold-change	3.96	2.12	1.15	2.26	3.92	2.15	7.23	1.55	0.73
	95% Confidence Interval	3.879–4.043	1.910–2.364	1.094–1.204	2.161–2.364	3.671–4.184	1.758–2.639	6.963–7.510	1.440–1.675	0.704–0.754
	*p*	0.009	0.031	0.010	0.005	0.000	0.000	0.000	0.021	0.012
37.5 µM CBD	Fold-change	3.75	2.74	1.78	3.02	3.23	4.39	15.31 .	2.06	0.69
	95% Confidence Interval	3.547–3.960	2.347–3.199	1.491–2.117	2.430–3.748.	2.712–3.853	3.070–6.280	12.934–18.121	1.575–2.694	0.644–0.744
	*p*	0.004	0.009	0.008	0.000	0.010	0.007	0.003	0.009	0.000
62.5 µg/mL TZB	Fold-change	6.11	4.21	1.66	3.02	5.16	2.69	14.24	2.32	1.021
	95% Confidence Interval	5.664–6.590	3.748–4.736	1.511–1.832	2.788–3.266	5.039–5.287	2.046–3.547	13.855–14.640	1.963–2.750	0.819–1.272
	*p*	0.005	0.015	0.000	0.026	0.000	0.011	0.000	0.015	0.758
18.75 µM CBD+ 62.5 µg/mL TZB	Fold-change	2.77	1.90	1.14	1.34	2.65	4.00	11.12	1.98	0.82
	95% Confidence Interval	2.532–3.028	1.575–2.299	1.086–1.196	1.212–1.480	2.532–2.769	3.049–5.251	9.890–12.497	1.858–2.117	0.786–0.848
	*p*	0.006	0.009	0.025	0.021	0.000	0.000	0.009	0.006	0.016
37.5 µM CBD+ 62.5 µg/mL TZB	Fold-change	2.33	1.67	1.31	1.66	1.61	2.81	11.83	1.92	0.78
	95% Confidence Interval	2.206–2.463	1.480–1.883	1.109–1.553	1.543–1.783	1.450–1.795	2.268–3.475	11.349–12.326	1.770–2.088	0.729–0.830
	*p*	0.007	0.004	0.000	0.000	0.013	0.000	0.000	0.004	0.023

Data are presented as mean fold-change relative to untreated control, normalized to GAPDH as the reference gene. Statistical analysis was performed using REST 2009 software with Pair-Wise Fixed Reallocation Randomization Test (2000 randomizations). Results are shown as mean fold-change with 95% confidence intervals from three independent biological replicates (*n* = 3). *p*-values < 0.05 were considered significant

*CBD* cannabidiol, *TZB* trastuzumab, *GRP78* glucose-regulated protein 78, *PERK* protein kinase RNA-like ER kinase, *IRE1* inositol-requiring enzyme 1, *ATF6* activating transcription factor 6, *eIF2A* eukaryotic initiation factor 2 A, *ATF4* activating transcription factor 4, *CHOP* C/EBP homologous protein, *XBP1s* spliced X-box binding protein 1, *HER2* human epidermal growth factor receptor 2

### CBD and TZB alone or in combination trigger apoptosis in SKBR3 cells

**Fig. 6:**
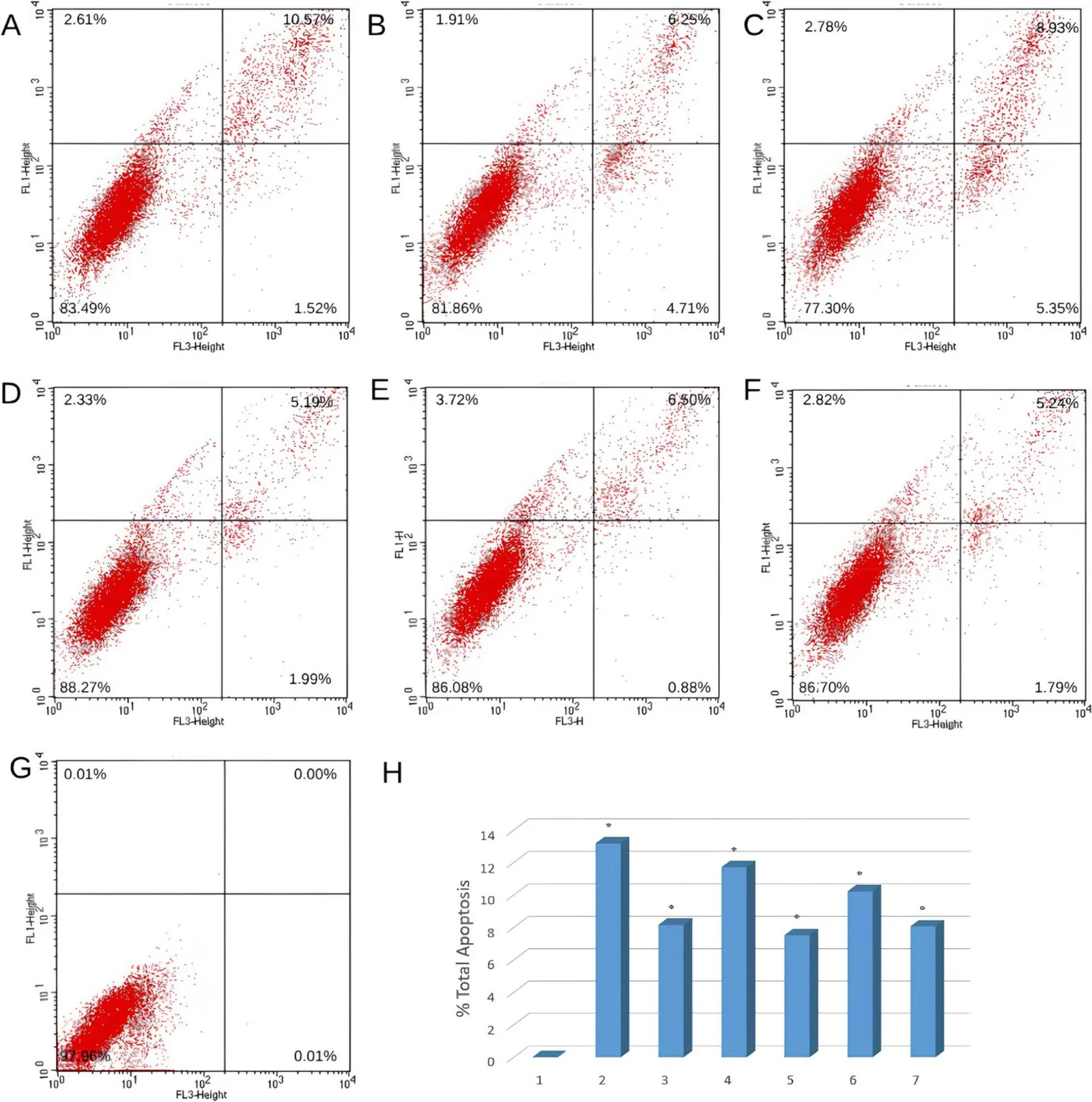
CBD, TZB, or their combination induces apoptosis in SKBR3 cells. SKBR3 cells were treated with CBD (9.38 µM, 18.75 µM, and 37.5 µM), TZB (62.5 µg/mL), and their combinations for 24 h. Apoptosis was detected using the Annexin V/PI apoptosis detection kit, and cells were analyzed by flow cytometry. **A**–**G** Representative dot plots of Annexin V-FITC versus PI staining for each treatment group: **A** 9.38 µM CBD, **B** 18.75 µM CBD, **C** 37.5 µM CBD, **D** 62.5 µg/mL TZB, **E** 18.75 µM CBD + 62.5 µg/mL TZB, **F** 37.5 µM CBD + 62.5 µg/mL TZB, and **G** Control. **H** Quantitative analysis of total apoptotic cells (early + late apoptosis). All treatment groups showed significantly higher percentages of apoptotic cells compared to the control group (all *P* < 0.001)

Treatment of SKBR3 cells with 9.38 µM, 18.75 µM, and 37.5 µM CBD reduced cell viability to 83.49%, 81.86%, and 77.30%, respectively, in a dose-dependent manner. The total percentage of apoptotic cells was measured as 13.18%, 8.16%, and 11.71%, respectively, at the same doses. We propose that the absence of a dose-dependent increase in apoptosis is likely due to the elevated proportion of necrotic cells, especially at CBD concentrations of 18.75 µM and 37.5 µM (all *P* < 0.001). Treatment of SKBR3 cells with 62.5 µg/mL TZB, 18.75 µM CBD + 62.5 µg/mL TZB, and 37.5 µM CBD + 62.5 µg/mL TZB reduced cell viability to 88.27%, 86.08%, and 86.70%, respectively. The total percentage of apoptotic cells was measured as 7.52%, 10.22%, and 8.06% at the same doses, respectively (all *P* < 0.001) (Fig. 6).

## Discussion

This study investigated the potential anticancer effects of CBD in the SKBR3 cell line, a HER2-positive aggressive breast cancer model. The effects of CBD and the monoclonal antibody TZB on the HER2 receptor, ER stress signaling pathway, and apoptosis were investigated, and this is the largest study on this subject to date in the literature. RTCA analysis demonstrated that following 72 h of treatment, significant antiproliferative activity was observed with 18.75 and 37.5 µM CBD alone, 62.5 µg/mL TZB alone, and their combinations. This antiproliferative effect was further correlated with the reduction in HER2 expression observed with CBD alone and in combination with TZB. Additionally, these doses significantly associated with upregulated the expression of key ER stress markers GRP78, PERK, IRE1, and ATF6, as well as their downstream targets eIF2A, ATF4, CHOP, and XBP1s. Flow cytometry analysis with Annexin V/PI staining further supported the cytotoxic effects of CBD, demonstrating increased apoptotic cell populations and reduced viable cell percentages.

The novel aspect of this study is the demonstration that CBD is associated with downregulation of HER2 gene expression in SKBR3 cells, and that this effect is maintained in combination with TZB. Furthermore, to the best of our knowledge, this is one of the few studies to systematically evaluate the association between CBD and TZB treatment and the induction of multiple ER stress signaling pathways in a HER2-positive breast cancer model, and to correlate these transcriptional changes with functional apoptotic outcomes.

The HER2 signaling pathway is activated via homodimerization or through heterodimerization with other family members, such as HER3 and EGFR, subsequently stimulating three main downstream signaling cascades: PI3K/AKT, MAPK, and phospholipase Cγ [44]. The PI3K/AKT pathway regulates cell survival, while the Ras/ERK1/2 and phospholipase Cγ pathways regulate cell proliferation [45, 46]. HER2 gene amplification or protein overexpression is detected in numerous solid tumors, including approximately 20% of breast and gastric cancers, as well as colorectal, lung, biliary tract, ovarian, and pancreatic cancers, and is commonly associated with aggressive disease, poor prognosis, and increased metastatic potential [47, 48]. Therefore, HER2 is recognized not only as a significant prognostic marker but also as a therapeutic target.

TZB, a monoclonal antibody used in the treatment of HER2-positive metastatic BC, has also become a therapeutic option for various solid tumors, including stomach, ovarian, non-small cell lung, and bladder cancers [49, 50]. The drug inhibits HER2 dimerization with other HER family receptors, leading to HER2 receptor internalization and degradation; thus, it suppresses activation of critical intracellular signaling pathways such as PI3K/Akt and MAPK [51]. The literature indicates that TZB disrupts HER2 dimerization [12] but has no effect on HER2 gene expression [52]. Consistent with these reports, our study found that 62.5 µg/mL TZB did not alter HER2 gene expression in SKBR3 cells but did significantly inhibit cell proliferation. A study using MCF-7/HER2, MDA-MB-453, BT-474, and SKBR3 cells showed that TZB at doses of 1.56-100 µg/ml inhibited proliferation, which is in agreement with our observations [6]. Although TZB is considered a relatively safe treatment option in HER2-positive BC [14], its clinical use is limited by various side effects, primarily cardiotoxicity [15, 16] and pulmonary toxicity [17], and the development of resistance [21–23]. Given these circumstances, there is a need for new therapeutic agents that can serve as alternatives or complements to current treatments.

CBD has emerged as a promising therapeutic agent in recent years for various cancer types, particularly breast cancer, although the mechanisms explaining its antitumor effect are not yet fully understood. Current findings suggest that these effects may largely occur through activation of CB1 and CB2 receptors and interaction with the endocannabinoid system [28, 29]. Studies in BC have reported that CBD reduces proliferation, invasion, metastasis and induces apoptosis in BC cell lines [36, 37, 42]. Studies with HER2-negative BC cell lines (SUM159, 4T1.2, MDA-MB231, MCF-7) have reported that CBD treatment at different doses ranging from 3 to 15 µM inhibits proliferation and migration [36, 37]. In a study conducted with HER2-positive BC cell lines (SKBR3 and BT-474), dose-dependent cytotoxic effects of CBD at 5, 10, 20, and 30 µM were observed [42]. In our study, we observed that 18.75 µM and 37.5 µM CBD were associated with reduced cell proliferation in a dose-dependent manner in SKBR3 cells, consistent with the findings of Kim et al. [42]. Notably, when combined with 62.5 µg/mL TZB, these CBD doses were associated with enhanced antiproliferative activity compared to either agent alone, particularly at the 18.75 µM CBD combination. One of the anti-proliferative mechanisms of CBD may be related to the HER2 receptor. In the above study conducted with HER2-positive SKBR3 and BT-474 cell lines, it was shown by Western blot and immunocytochemistry staining that 20 µM CBD treatment was associated with a decrease in HER2 protein levels [42].

In our study, we observed that CBD was significantly associated with decreased HER2 gene expression at the mRNA level in SKBR3 cells. This decrease was observed with 9.38 µM, 18.75 µM, and 37.5 µM CBD alone, as well as in combination with TZB (18.75 µM CBD + 62.5 µg/mL TZB and 37.5 µM CBD + 62.5 µg/mL TZB). Importantly, the reduction in HER2 expression in the combination groups appeared to be primarily consistent with the effect of CBD, as TZB alone did not alter HER2 expression. Our gene expression data are consistent with and support the protein-level findings reported by Kim et al. [42], suggesting that CBD downregulates HER2 expression at both transcriptional and translational levels.

In addition to the effects of CBD and TZB on HER2, another mechanism of action suggested in recent years is via the ER stress signaling pathway. Although there are some reports in the literature regarding TZB and ER stress, they are limited and not directly related to the involvement of ER stress signaling pathways. A study reported that the combination of tunicamycin, an ER stress inducer, and TZB synergistically inhibited proliferation and increased apoptosis in HER2-positive BC cell lines (MCF-7/HER2, SKBR3, and BT-474) [6]. Our findings extend this observation by demonstrating that 62.5 µg/mL TZB treatment alone is significantly associated with increased expression of multiple ER stress markers, including GRP78, PERK, IRE1, ATF6, eIF2A, ATF4, CHOP, and XBP1s, in SKBR3 cells. This transcriptional upregulation was accompanied by reduced cell viability and increased total apoptosis, as determined by RTCA and Annexin V/PI flow cytometry, respectively. These data suggest that induction of ER stress-mediated apoptosis may represent a previously underappreciated mechanism of TZB action, beyond its well-established effect on HER2 dimerization.The majority of studies on the anticancer effect of CBD through the induction of ER stress in the literature are more prevalent in tumors other than BC [30–34]. A study using glioma cells reported that CBD treatment at doses ranging from 10 to 50 µM triggered ER stress by increasing eIF2A and ATF4 protein levels in a dose-dependent manner [30]. In a study conducted with cholangiocarcinoma cells, 30 µM CBD treatment was reported to induce ER stress-mediated apoptosis by increasing the expression levels of p-PERK, BiP, ATF4, CHOP, and BAX [31]. Similarly, in a study using an ovarian cell line, treatment with 30 and 50 µM CBD was shown to trigger ER stress-induced apoptosis by upregulating the gene expression levels of GRP78, ATF4, XBP1, and CHOP [31, 32]. In a study using hepatocellular carcinoma cells, CBD treatment was reported to increase CHOP and XBP1s gene expression and protein levels, particularly at 40 and 50 µM [34].

In studies using CBD in BC lines, treatment with 20 µM CBD for 48 h did not alter the viability of the non-tumorigenic MCF10A cell line, whereas it significantly reduced cell viability in HER2-negative MCF-7 and MDA-MB-231 cells. In MCF-7 cells, CBD was suggested to activate ER stress and the UPR by increasing GRP78 and ATF4 protein levels [35]. It has been suggested that treatment with 5 µmol/L CBD in MDA-MB-231 cells induces ER stress and reduces viability by increasing p-eIF2A protein levels [41]. In the literature, apart from the mentioned studies investigating CBD-induced ER stress mechanisms in BC, research examining the molecular pathways regulating this process in the HER2-positive BC subtype remains limited. In a study by Shrivastava et al., treatment of SKBR3 cells with 0–10 µmol/L CBD for 24 h reduced cell viability in a dose-dependent manner [41]. In a more recent study, treatment with 5, 10, and 20 µM CBD for 24 h increased ATF4 and CHOP protein levels in SKBR3 and BT-474 cells [42]. Our study significantly expands upon these findings by demonstrating that CBD treatment at 9.38 µM, 18.75 µM, and 37.5 µM, as well as in combination with TZB, is associated with upregulation of all three major ER stress sensor pathways (PERK, IRE1, and ATF6) at the transcriptional level in SKBR3 cells. This comprehensive activation of multiple ER stress branches, coupled with increased expression of downstream pro-apoptotic markers (ATF4, CHOP, XBP1s) and confirmed by Annexin V/PI flow cytometry, provides strong evidence consistent with the hypothesis that ER stress-mediated apoptosis is a key mechanism underlying the antiproliferative effects of CBD and its combination with TZB in HER2-positive breast cancer cells.In the literature, it has been suggested that TZB disrupts HER2 dimerization, thereby suppressing receptor signaling [12], whereas CBD has been reported to bind with high affinity to HER2 tyrosine kinase [53] and to exert a downregulatory effect on HER2 expression, leading to reduced receptor density on the cell surface [42]. In the present study, we observed that TZB did not affect HER2 expression, while CBD decreased HER2 expression. Consistently, in the combination treatment, we detected a reduction in HER2 expression that appeared to be consistent with the effect of CBD, in line with our findings. Following its binding to HER2, TZB is internalized and metabolized into peptides and amino acids within the cell, which are subsequently eliminated by the reticuloendothelial system [54]. In contrast, CBD is primarily metabolized in the liver by cytochrome P450 mixed-function oxidases [55]. The fact that these two agents are metabolized via different pathways raises the possibility that combinations of CBD and TZB at lower doses could offer enhanced efficacy and reduced side effects, presenting a therapeutic potential for the treatment of HER2-positive BC.

This study presents functional and transcriptional evidence that CBD, TZB, and their combination are associated with ER stress and trigger apoptosis in HER2-positive breast cancer cells; however, it also harbors certain methodological and translational limitations. First, the activation levels of key pathways in the ER stress response and changes in HER2 expression were not directly validated at the protein level, thereby limiting the correlation between the observed transcriptomic changes and functional outcomes. Second, although our findings are consistent with previous literature demonstrating that CBD reduces viability in a dose-dependent manner, suppresses HER2 protein levels, and decreases ATF4 and CHOP protein levels in BT-474 cells [42], the study was conducted using only a single HER2-positive cell line SKBR3. The lack of validation in additional HER2-positive cell models limits the generalizability of the results. Third, the absence of trastuzumab-resistant HER2-positive models represents a significant limitation, as our findings are currently restricted to trastuzumab-sensitive SKBR3 cells; therefore, the translational relevance of the combination therapy in clinically relevant settings where trastuzumab resistance commonly develops remains to be established. Fourth, the effects of CBD on non-malignant breast epithelial cells were not evaluated in this study. Nevertheless, previous studies have shown that CBD at concentrations up to 20 µM does not exert significant cytotoxic effects on MCF-10A cells [35], suggesting a potential tumor-selective profile, although direct comparison within the same experimental setting remains a limitation. Fifth, although our data show a strong correlation between ER stress marker upregulation and apoptosis, we have not performed loss-of-function experiments (e.g., pharmacological inhibition of ER stress pathways or CHOP knockdown) to establish causation. Thus, the observed relationship is associative rather than conclusively causal, although it is consistent with the established role of the PERK-ATF4-CHOP axis in ER stress-mediated apoptosis [43, 44]. Future mechanistic studies are warranted to confirm this causal link. Furthermore, the CBD concentrations used in our study are difficult to achieve systemically in humans. Although intraperitoneal administration of CBD has been reported to exert antitumor effects in xenograft models [56], this route does not reflect human therapeutic regimens; further studies are required before clinical application.

## Conclusion

In conclusion, both TZB and CBD are associated with ER stress-mediated apoptosis. While TZB exerts its effect by disrupting HER2 dimerization, CBD is associated with the inhibition of this signaling pathway in conjunction with the downregulation of HER2 gene expression. Our findings suggest that CBD may have potential as a therapeutic agent in HER2-positive breast cancer; however, further preclinical and clinical studies are warranted to establish its efficacy and safety as a monotherapy. Additionally, our results provide a basis for future studies investigating lower-dose combination regimens of CBD and TZB, which may offer a strategy to reduce potential side effects.

## Data Availability

All data generated or analysed during this study are included in this published article.

## Abbreviations

ATF4: Activating Transcription Factor 4
ATF6: Activating Transcription Factor 6
BC: Breast Cancer
CBD: Cannabidiol
CHOP: C/EBP Homologous Protein
eIF2A: Eukaryotic Translation Initiation Factor 2α
ER: Endoplasmic Reticulum
GRP78: Glucose-Regulated Protein 78 kDa
IRE1: Inositol-Requiring Enzyme 1
PERK: PRKR-like ER Kinase
RTCA: Real-Time Cell Analyzer
TZB: Trastuzumab
UPR: Unfolded Protein Response
XBP1s: Spliced X-Box-Binding Protein 1
